# An ultra-high bandwidth nano-electronic interface to the interior of living cells with integrated fluorescence readout of metabolic activity

**DOI:** 10.1038/s41598-020-67408-5

**Published:** 2020-07-01

**Authors:** Dandan Ren, Zahra Nemati, Chia-Hung Lee, Jinfeng Li, Kamel Haddadi, Douglas C. Wallace, Peter J. Burke

**Affiliations:** 1 Department of Electrical Engineering and Computer Science, University of California, Irvine, CA 92697 USA; 2 Department of Materials Science and Engineering, University of California, Irvine, CA 92697 USA; 3 Department of Biomedical Engineering, University of California, Irvine, CA 92697 USA; 4 Department of Physics and Astronomy, University of California, Irvine, CA 92697 USA; 5 CNRS, UMR 8520, Institute of Electronics, Microelectronics and Nanotechnology (IEMN), University of Lille, 59000 Lille, France; 6 Center for Mitochondrial and Epigenomic Medicine, Children’s Hospital of Philadelphia and Department of Pediatrics, Division of Human Genetics, University of Pennsylvania, Philadelphia, PA 19104 USA; 7 Department of Chemical and Biomolecular Engineering, University of California, Irvine, CA 92697 USA; 8 Chemical and Materials Physics Program, University of California, Irvine, CA 92697 USA

**Keywords:** Bionanoelectronics, Nanobiotechnology

## Abstract

We present the first ever broadband, calibrated electrical connection to the inside of a cell. The interior of a vital, living cell contains multiple dynamic and electrically active organelles such as mitochondria, chloroplasts, lysosomes, and the endoplasmic reticulum. However, little is known about the detailed electrical activity inside the cell. Here we show an ultra-high bandwidth nano-electronic interface to the interior of living cells with integrated fluorescence readout of metabolic activity. On-chip/on-petri dish nanoscale capacitance calibration standards are used to quantify the electronic coupling from bench to cell from DC to 26 GHz (with cell images at 22 GHz). The interaction of static to high frequency electromagnetic fields with the cell constituents induce currents of free charges and local reorganization of linked charges. As such, this enables a direct, calibrated, quantitative, nanoscale electronic interface to the interior of living cells. The interface could have a variety of applications in interfacing life sciences to nano-electronics, including electronic assays of membrane potential dynamics, nano-electronic actuation of cellular activity, and tomographic, nano-radar imaging of the morphology of vital organelles in the cytoplasm, during all phases of the cell life cycle (from development to senescence), under a variety of physiological environments, and under a broad suite of pharmacological manipulations.

The interior of a vital, living cell contains multiple dynamic and electrically active organelles such as mitochondria, chloroplasts, and lysosomes. However, little is known about the detailed electrical activity inside the cell, in spite of well-recognized significance in biology and medicine. For example, the membrane potential of mitochondria “flickers” with a pattern that depends on organism age in model organisms such as C Elegans^1^. Why does the dynamic electronic properties of this organelle relate to aging? In another example, the nano-scale morphology of mitochondria during apoptosis changes in a controversial way during chemotherapy and natural cell death^2^. How does the mitochondrial morphology change during programmed cell death (apoptosis)^3,4^? The key to answering these and other similar questions requires an electronic interface to vital, living cells in order to unlock the scientific mysteries and provide actionable medical treatments for many of the modern maladies plaguing human society such as aging, cancer, diabetes, and neuro-degenerative disorders, all of which are related to the internal electronic activity of cell organelles and biology. But how can we enable such a broadband and calibrated electronic interface to cells?

We and others^5–9^ have recently been working on calibrated, ultra-high frequency imaging of nano-electronic components such as nanowires, nanotubes, quantum dots, etc. as a method to characterize the electronic properties of nanoelectronic and beyond Moore’s law CMOS type components at frequencies from RF all the way into the mm-wave, such as frequencies required for 5G. Because at high frequencies, every mm of wire matters, it is important to calibrate the entire electronic system, and for this purpose we developed a capacitance calibration that enables a vector, broadband, nano-electronic calibration from DC to over 20 GHz. This has been adopted in industry and has been commercialized by several companies (e.g. Keysight Technologies, Prime Nano, MC2-Technologies). This on-chip calibration enables complete quantitative determination of the coupling from rack of electronics to the tip of an AFM probe, a metal wire with final diameter of order 10 nm. Because different microwave frequencies penetrate different depths, this technique has even been demonstrated^10–12^ (using multiple frequencies) to image the metal traces buried deep inside of a semiconductor wafer, which is not imaged using surface AFM techniques, since the traces are buried inside of the wafer. This raises an interesting possibility: If one can non-invasively image inside of semiconductor wafers with nanoscale resolution, can one do the same to peer inside of living cells?

We and others^13–21^ have recently used this technique to image the cytoplasm of fixed cells and subcellular organelles. Recently, we demonstrated^22^ for the first time capacitive nano-electronic imaging vital isolated mitochondria at 7 GHz. However, in that work, we had to separate the mitochondria into two aliquots and the vitality was not confirmed during the nano-electronic imaging due to the opaque nature of the substrate. Furthermore, the calibration was not quantitative because the stray parasitic capacitance of the probe connections was not quantified. The reason is that the calibration standards were on a different wafer than the biological system we were measuring (in our case vital mitochondria isolated from living cells), and the stray electronic capacitance are not quantified in that case.

In this work, we combine many of the techniques that electrical engineers in nanotechnology have been developing for years with “industry standard” biotechnology probes of cell vitality and metabolic activity probed with fluorescence microscopy to demonstrate an ultra-high bandwidth nano-electronic interface to the interior of living cells with integrated fluorescence readout of metabolic activity. A preliminary report of this work was presented at a conference in September 2019^23^. Several key advances are demonstrated in this work that enables such a system: First, we take a major step forward and extend the calibration technique that we and others have developed for dry systems and show it functions in liquid imaging conditions, where the liquid is physiological buffer. Because the buffer is very conducting^24,25^, we were surprised and delighted when our experiments confirm that the technique we have developed over the past 10 years in dry works in liquid. It is not obvious that is should work, since the “shielding”, conductivity, and frequency dependence of the liquid creates challenging, additional parasitic electrical elements that need to be calibrated out. Although we do not have a detailed model for the effect of liquid in this system, we do demonstrate that we can successfully image capacitances of order fF (10^−15^ F) in liquid, and we even provide some data on the additional stray electrical effects of the conducting liquid on this technique. Second, we integrated the calibration capacitance standards “on chip/on-petri dish” so that imaging of cultured cells is done in the same field of view as capacitive imaging of the calibration disks. This enables us to quantitatively calibrate the cell image as a conductance and capacitance image, over a broad frequency range, from DC to over 20 GHz. (This is the frequency domain analog of time-domain, as such it is a “nano-radar” of cells.) Third, we use a transparent electrical ground (ITO) to enable back side, simultaneous fluorescence imaging. The microscope objective is only 100 microns from the sample and an oil-immersion 100× objective can be used. This simultaneous optical imaging capability is fully compatible with all of the best modern optical techniques, such as confocal, STED, Palm, Airy, etc. and is limited only by the budget of the lab. While we cannot demonstrate all of those in one paper, in this work we do demonstrate simultaneous fluorescence imaging of vital cellular properties (mitochondrial membrane potential). This provides “proof of life” which shows the nano-electronic interface is actually in contact with living cells, and is a key advance of this work, since prior work (including our own^22^) was “blind” to the cellular vitality. Fourth, and finally, instead of using complicated resonator circuits to boost electrical sensitivity, as we and others have done in the past^5–7,13,22^, we show that with careful attention to elementary microwave engineering principles, that such a resonator circuit is not necessary and the commercial off the shelf (COTS) microwave vector network analyzers (VNAs) are completely suitable for probes of this type, thus enabling broadband, frequency dependent behavior to be probed at frequencies up to and beyond 20 GHz. Although each of these advances could be its own separate paper, the combination of all of them effectively defines a powerful new way to probe living systems using nanoelectronics. As such, this enables a direct, calibrated, quantitative, nanoscale electronic interface to living cells (Fig. 1).

**Figure 1:**
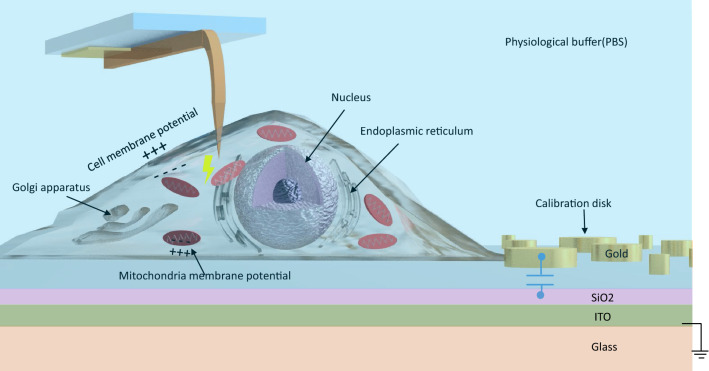
An ultra-high bandwidth nano-electronic interface to living cells with integrated fluorescence readout of metabolic activity.

Figure 2 shows the key components of our system: A microwave network analyzer (Keysight Model # N5222A PNA VNA) sends a microwave signal (swept from 10 MHz to 26.5 GHz) down a coaxial cable. The end of the coaxial cable is connected to a sharp, metal AFM tip (Rocky Mountain Nanotechnologies #12Pt400A, 0.3 N/m), which presents a very high impedance to the incoming microwave, thus reflecting some of the microwave back up into the network analyzer. The network analyzer measures the (complex, frequency dependent) microwave reflection coefficient *S*_11_, defined as the complex ratio (in both magnitude |*S*_11_| and phase-shift θ) between the reflected and incident microwave signals. As the tip is scanned, the tip-ground impedance changes and thus *S*_11_ changes. The system then creates an image of *S*_11_ vs position (x,y). In an ideal system, the reflected microwave reflection coefficient (*S*_11_) is related to the complex impedance of the tip-sample Z_tip_ through the standard equation: S_11,tip_ = (Z_tip_ − 50 Ω)/(Z_tip_ + 50 Ω), which can be inverted to find Z_tip_. The equivalent electrical circuit at the probe tip (Z_tip_ = R + jX), consists of mainly the capacitance C between the tip and the ground plane (X = − 1/(C2πf)), and this changes as the tip is scanned. Thus, a map of the tip-sample impedance (which behaves mostly as a capacitor) can be obtained with high spatial resolution. An Olympus IX71 inverted light microscope (ILM) is attached to image the sample from the bottom, and the system is installed inside a shielded Faraday cage situated on an anti-vibration table. A broad-spectrum fluorescence illumination source X-Cite 120 PC Q is connected to the inverted light microscope along with TRIC and FITC filters. In our case, a customized filter block for TMRE fluorescence, described in more detail below, is used to assay the mitochondrial membrane potential, a common metabolic assay. While the microwave network analyzer and AFM are commercial off the shelf systems, and the Olympus microscope is also, we had to have a custom adapter fitted to integrate the two separate systems together.

**Figure 2:**
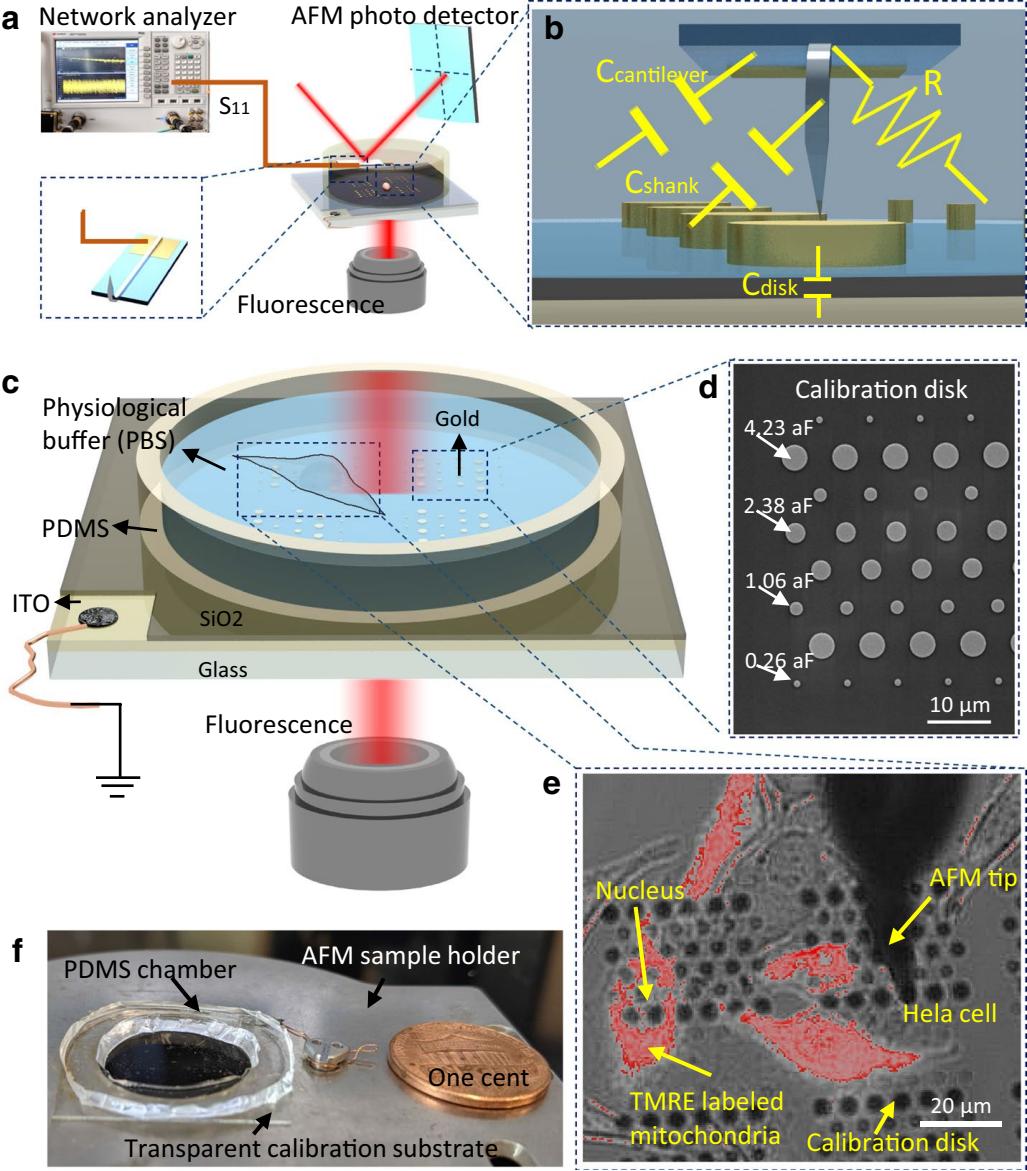
(**a**) Microwave vector network analyzer (Keysight N5222A PNA) measures the microwave signal reflected by a sharp, metal AFM tip through a microwave coaxial cable. A standard AFM scanner is used to move the AFM tip over the sample being studied. (**b**) The electrical equivalent circuit at the end of the cable consists of mainly the capacitance between the tip and the ground plane, and this changes as the tip is scanned. However, there are extra unwanted (“parasitic”) circuit elements, which are assumed to be constant as the tip is scanned, but which nevertheless need to be calibrated out for a true calibrated image. (**c**) Sample chamber showing live cells and calibrated standard disks, as well as the optically transparent electrical ground plane (ITO). (**d**) SEM image of the calibration disks. (**e**) Photograph of the sample chamber. (**f**) Superimposed brightfield and fluorescence image of vital HeLa cell culture on the disk. The fluorescence label is TMRE which is indicative of the mitochondrial membrane potential.

Figure 3 shows an image of the (raw, uncalibrated) magnitude and phase-shift of the microwave signal measured by the network analyzer as the tip is scanned over metal disks of various diameters between 1 and 4 µm for several frequencies between 1 and 26 GHz, with a power of − 30 dBm and resolution bandwidth of 0.5 kHz. The temporal resolution of the measurement is the inverse of the resolution bandwidth, i.e. 2 ms, per pixel. We scanned slower than this to avoid transients. However, note this can be improved with lower noise electronics. The noise figure of a typical VNA is about 100,000 K, and typical low noise amplifiers have noise temperatures of order 100 K. Therefore, by using an LNA, the temporal resolution could be 2 micro seconds at the same signal to noise. Note, we have not optimized the signal to noise or temporal resolution in this setup. The contrast is due to the change in the capacitance between the tip and the ground plane as the tip is scanned. Although this clearly demonstrates our ability to image capacitance over a broad frequency range with this system, we were not satisfied and thus turned our attention to a more refined calibration, discussed next.

**Figure 3:**
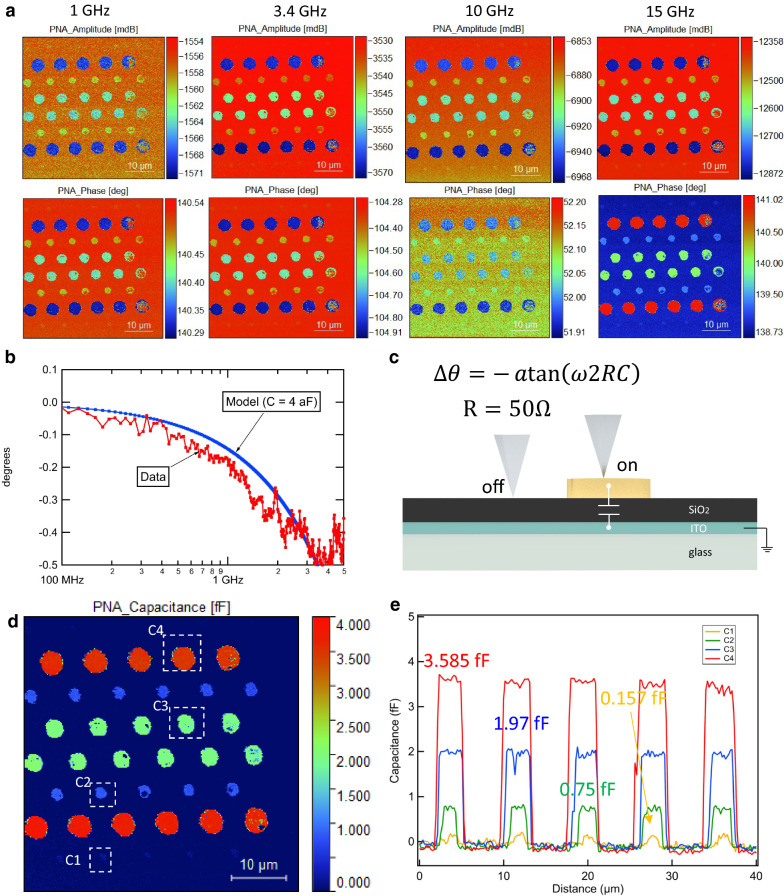
Scanning probe microscopy measurement results on calibration disk in air. (**a**) Measured S_11_ amplitude and phase-shift images at four different frequencies (1 GHz, 3.4 GHz, 10 GHz and 15 GHz). (**b**, **c**) Expected broadband frequency dependent S_11_ phase change of a 4 fF capacitor and measured phase change C4 calibration disk. (At frequencies higher the 5 GHz additional resonances due to the finite length of the wire used in the probe show up.) (**d**) Calibrated capacitance image from measured S_11_ at 3.4 GHz. (**e**) Calibrated capacitance profile obtained from (**d**) which shows a consistent relative capacitance value between C1, C2, C3 and C4.

A quantitative calibration is challenging due to the multiple non-idealities in the system, such as transmission losses and phase-shift in the coaxial cabling, mismatching effects inherent to the different connectors, and (most importantly) parasitic capacitance (or more generally impedance) between the short length of wire connecting the probe tip to the coaxial cable and ground (Fig. 2a). This parasitic impedance is in parallel with the tip-sample capacitance of interest. A very reasonable assumption is that these systematic errors are constant as the tip scanned, and therefore, with sufficient effort, that they can be measured and subtracted from the measured data to determine only the tip-sample (e.g. tip-cell) capacitance arising from fields only in the immediate vicinity of the sharp tip as the tip is scanned, with resolution determined by the tip sharpness and spreading of the fields, of order 10 nm. In order to measure these non-idealities (which include all of the systematic errors listed above), we employ an on-chip set of calibration standards, described next in detail.

The non-idealities of the system can be described mathematically as a 2×2 complex, frequency dependent “error” matrix e, which causes the measured S_11measured_ by the vector network analyzer to be related to the S_11,tip_ at the apex tip by: S_11measured_ = e_00_ + S_11_e_01_/(1 − S_11tip_e_11_). (Mathematically, the complex terms e_00_, e_01_ and e_11_ are complex calibration coefficients that correspond respectively to the directivity, source match and reflection tracking errors.) The three (complex, frequency dependent) elements e_00_, e_01_ and e_11_ can be determined at each frequency by using known standards for S_11tip_, recording S_11measured_, and solving for e_00_, e_01_ and e_11_. At least three calibration standards with known *S*_11,tip_ are required since there are three unknowns. Once the e matrix is determined, future S_11measured_ can be converted to S_11,tip_ computationally by inverting the above formula, and then Z_tip_ by inverting S_11,tip_ = (Z_tip_ − 50 Ω)/(Z_tip_ + 50 Ω). In this way, a calibrated, quantitative image of the impedance between the tip and the sample can be obtained. We and others^5–9^ have developed a suite of nanoscale metal (MOS) capacitors with very well-known absolute capacitance values dependent only on the disk geometry as “standards” to effectively determine all the systematic errors in the system. However, these commercial nanoscale capacitance standards developed by some of us (Lille) and commercialized by Keysight/MC2-Technologies (a spinoff from Univ. of Lille) are optically opaque and therefore not compatible with our imaging system.

In order to enable simultaneous capacitance calibration and fluorescence imaging, we designed and fabricated our own capacitance calibration standards to enable simultaneous fluorescence imaging (Supplementary information 1). The technical requirement of a transparent substrate is needed to obtain fluorescence signals which we solved by using a glass substrate. However, a ground plane is needed, and it must be within 100 nm of the metal disks to provide a large enough capacitance to calibrate the system. Therefore, we used ITO with a dielectric layer of 100 nm SiO_2_ and the disks on top. The detailed clean room fabrication process is shown in Supplementary information 1. The capacitance disks vary from 1 to 8 microns in diameter, and the theoretical capacitance values vary from 1 to 17 fF. In Fig. 3, we show how it works on these new transparent ground plane based substrate: The (calibrated) capacitance image demonstrated the procedure is successfully applied in our new generation calibration wafers. In separate experiments we have also confirmed that the Keysight/MC2-Technologies calibration standards and our own calibration standards give comparable results.

In prior work we and others used resonators to enhance the contrast of the image^5–7,13,22^. However, in this work, no resonator is used, and the image contrast is fine. At the power levels and averaging time used in this work (− 30 dBm source power in air, − 10 dBm in liquid, resolution bandwidth 0.5 kHz in air, 1 kHz in liquid), the capacitance standard signal was clearly visible in the images, indicating the signal to noise ratio is sufficient to resolve capacitances of order fF (10^−15^ F). (A detailed noise analysis will be presented in a future publication.) Although we defer to the Supplementary information 1 for a detailed discussion of narrowband vs broadband sensitivity, in this (broadband) work, we can also sweep the frequency and measure the reflection coefficient *S*_11m_ on and off the disk to confirm that the impedance is mostly capacitive. Figure 3 shows the change in phase-shift vs. frequency for on vs off disk. It can be shown using equations above that for small C, a capacitive load would give △θ = − *atan*(2πf × 2 × 50 C), (see Supplementary information 1 for Taylor expansion in C), and that is indeed what is observed, further confirming the capacitive model of the calibration.

The calibration procedure attempts to correct for all parasitic capacitances and conductances from tip to ground that do not change as the tip is scanned. However, this background can be much more severe in liquid due to the absorbing properties of salt rich physiological buffer at RF and microwave frequencies. Up until our work, no group has been able to do a broadband study of the significance of this excessive loss on the ability to resolve small features and capacitance in liquid. (Previous work including ours and others have focused exclusively on narrowband resonant conditions^24,25^.) The difference between the reflection off the tip in Fig. 3c in liquid vs. air is the first quantitative broadband measurement of the effect. It shows that, while significant, it does not completely mask the signal from the calibration capacitance disks. This proves for the first time that the imaging of nanoscale capacitances in physiologically relevant liquid is possible and even can be done quantitively.

That being said, we can actually quantify the liquid absorption using the data in Fig. 3c. The liquid absorbs about 5 dB more than just air, indicating that the impedance (resistive) is of order 100 ohms. We would like to note that because of the significant loss, our accomplishment (below) of getting the nanoelectrode into the interior of the cell is important, since it enables electrical sensing and actuation at frequencies where strong attenuation would prevent such interfacing due to the nanometer scale Debye screening length, as was clearly demonstrated (in theoretical simulations only) in Ref.^26^.

An additional source of uncertainty is the crosstalk (Ref.^13^), that is, stray capacitance between tip and ground that changes as the tip is scanned due to vertical movement of the tip. The signal to noise is NOT an indicator of the crosstalk, as noise can be present even if crosstalk is insignificant. This crosstalk is not likely to be the cause of the low contrast in liquid between disk and non-disk regions of the substrate, as the tip does not change its height very much as it moves over the disk. However, for the cell imaging, it will be an important issue for determining the final spatial resolution of the technique, as well as the tip shape and geometry, as was shown in Ref.^13^. For this reason, in the future it will be important to developed improved tip designs (e.g.^27^) which minimize the crosstalk. None of this discussion detracts from the main point of this paper, which is quantitative coupling over a broadband frequency range to a living cell in physiological liquid with on chip capacitance calibration standards. We turn next to the imaging in liquid. Figure 4 shows an image of the (raw, uncalibrated) magnitude and phase-shift of the microwave complex reflection coefficient measured by the network analyzer as the tip is scanned for several frequencies between 1 and 22 GHz in liquid (140 mM KCl). The images clearly show the contrast even in spite of the extra parasitic circuit properties of the conducting liquid. This clearly demonstrated that our system, in spite of the extra parasitic of the liquid, can image capacitances down to a few fF up to over 10 GHz in physiologically relevant liquid buffer.

**Figure 4:**
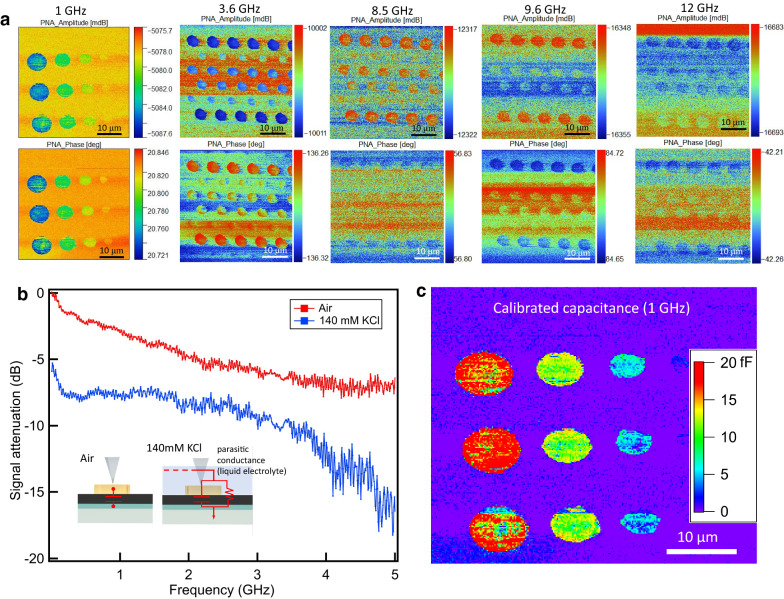
Scanning microwave microscopy measurement results on calibration disks in salt solution. (**a**) Measured S_11_ amplitude and phase-shift images at five different frequencies (1 GHz, disk diameters are 2 um, 4 um, 6 um and 8 um; 3.6 GHz, 8.5 GHz, 9.6 GHz and 12 GHz, disk diameters are 1 um, 2 um, 3 um and 4 um). Note the signal to noise varies from experiment to experiment and, although it has not been studied in detail, does not depend in general on frequency. (**b**) Broadband frequency dependent S_11_ amplitude at the end of a hanging tip in air (red) and 140 mM KCl (blue). (**c**) Calibrated capacitance at 1 GHz.

The calibration procedure in principle can be used again to determine the parasitic circuit elements introduced by the liquid and subtract them off of the measurement, to give a calibrated capacitance image. This is done in Fig. 4. The procedure provides a calibrated impedance measurement, although the calibrated capacitance is not perfect. For example, the parasitic impedance brought by the liquid depends on the liquid electrical properties and also the liquid thickness. This latter may vary during the measurement process, especially as the measurements done in air are subject to evaporation.

In order to further determine the effect of the liquid, one can look at the magnitude of S_11measured_ vs. frequency for the case of with vs. without liquid (Fig. 4). Without the liquid, the magnitude of the signal (|S_11measured_| less than 0 dB) is mostly due to transmission losses in the coaxial cable, which are up to 3 dB. With liquid, the same measurement gives about 5 dB of additional loss due to the liquid. In conclusion, while the liquid provides significant additional absorption of the microwaves (as expected from our previous studies^24,25^), it does not completely mask the image, and we are able to successfully, for the first time ever, image fF scale capacitances at GHz frequencies in physiological relevant solution. Using this system, we turn next to imaging biological systems.

The oxide surface of the glass calibration wafers is a perfect substrate for cell culture and immobilization. We have two tracks for imaging HeLa cells in this system: fixed (for better more stable images) and live (for more real time biological response measurements). The cell culture and immobilization procedures are standard and described in detail in Supplementary information 1. For the metabolic activity, the HeLa cells were tagged with 100 nM TMRE potentiometric dye in order to image the mitochondrial membrane potential distribution within the cell.

The HeLa cell preparation protocol is shown in Supplementary information 1, and include protocols for imaging live cells and fixed cells. In Fig. 5a, b, two images are shown of cultured, live cells. The first is a brightfield image (Fig. 5a) showing also the calibration capacitors (disks) and the conductive AFM tip. While the cells are visible, one does not know if they are alive just from the brightfield image. Since there is temperature shock upon transferring them to the microscope (we do not yet have an environmental chamber), the cells begin to die soon after the chamber is loaded to start imaging. Figure 5b is a fluorescence image with TMRE showing mitochondrial membrane potential. Some cells are in the process of dying and have low membrane potential; some are dead already. This illustrates the necessity to image fluorescently simultaneously, otherwise one does not know if the cells are alive or dead. (There is one other preliminary report on live cells^20^, but the cell vitality was not confirmed by fluorescence as we have done here.) This clearly demonstrates the motivation for this work. The cells can even be mechanically manipulated, which could be an interesting avenue to look at mechanical electrical coupling in cellular organelles. Although the system is demonstrated to image live cells via fluorescence, we used fixed cells as a first step to demonstrate the in-situ/on-petri dish calibration in liquid. Figure 5c–e shows various optical images of fixed HeLa cells. Figure 5c, d shows fluorescence images of fixed HeLa cells tagged with MitoTracker green. MitoTracker green tags mitochondrial regardless of their membrane potential. The mitochondria are clearly visible around the nucleus. Figure 5e shows a brightfield image another fixed HeLa cell which we decided to image with our microwave system. Figure 5f shows the magnitude image of the microwave reflection coefficient *S*_11measured_ of the fixed HeLa cell imaged in Fig. 5e, with integrated capacitance standards visible in the image. We have used these capacitance standards to calibrate the image, and the resultant calibrated capacitance at 22 GHz is shown on the bottom right of Fig. 5g. This clearly demonstrates the ability to calibrate nanoscale capacitance images in situ of biological structures.

**Figure 5:**
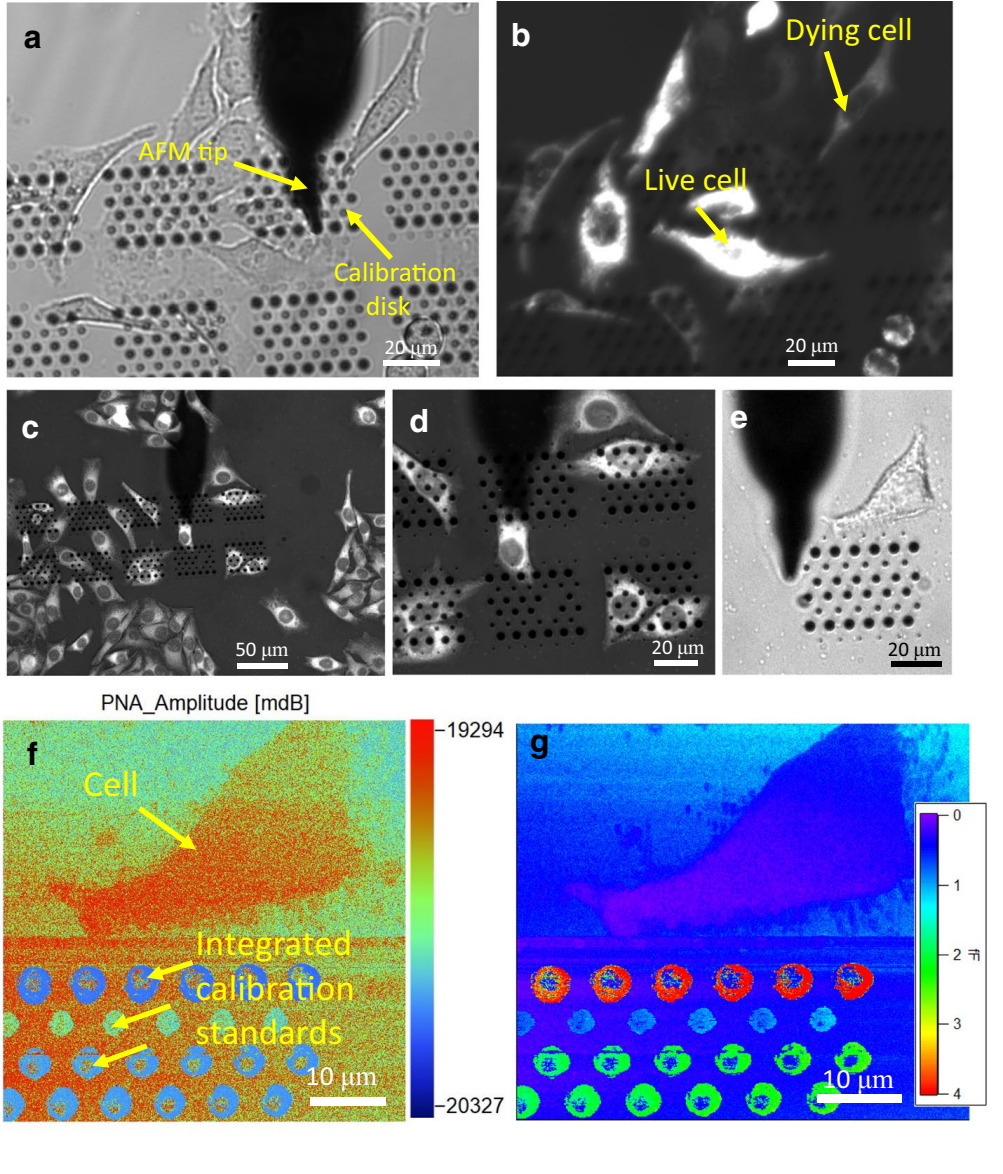
(**a**) Live HeLa cells imaged via brightfield which shows the integrated nanoscale calibration capacitors and AFM tip. (**b**) Same live HeLa cells, labeled with TMRE, simultaneously imaged via fluorescence. The TMRE signal intensity corresponds to cell viability, as it indicates the mitochondria are breathing, consuming oxygen, creating ATP, and sustaining a membrane potential. (TMRE is a membrane sensitive dye.) Note that both live and dead cells appear the same in a but not b, emphasizing the fact that any new nano-probe on cells must have separate confirmation, or “proof of life”, before any conclusions can be drawn about the biology in real time. (**c**, **d**) Fluorescent images of fixed HeLa cells labeled with Mitotracker Green, which labels mitochondria regardless of their membrane potential. The mitochondrial dense region around the nucleus, AFM tip, and calibration disks are clearly visible. (**e**) A single fixed HeLa cell in brightfield view on a set of calibration disks. (**f**) Amplitude of the complex reflection coefficient image of the fixed HeLa cell in (**e**) of a 22 GHz microwave signal, with integrated nanoscale calibration capacitors of value 1,2, and 4 aF clearly visible in the same image. (**g**) Calibrated capacitance of image (**f**) (see main text), demonstrating a quantitative, high bandwidth interface compatible with physiological buffer.

What is the physical mechanism underlying the intracellular gradient of capacitance shown in Fig. 5g? Because these are the first calibrated, broadband measurements of cells, there is no theoretical model that describes the capacitance. In addition, crosstalk (discussed above) may be more significant since the cell height is larger than that of the disks. However, we still resolve structure in the capacitive imaging, which clearly indicates that cross talk is not the dominant component of the signal, even in the cell case. Developing a biophysical model of the capacitance density (and frequency dependent dielectric constant) at the nanoscale level for living cells is beyond the scope of this paper. However, without data, there is no point to make up models, so we have provided the very first broadband data that will be the basis and foundation for these future dielectric models. In order to address the achievement of the present work, we have performed a series of experiments on live cells. In so doing, we have found the following: First, the tip spring constant is quite strong, and the cell is soft, so that SMM imaging of live cells is not suitable for soft tissue without significant perturbation of cell morphology. In 100% of over 60 experiments on three cell lines and multiple substrates with differing surface chemistry (see Supplementary information 1), our AFM probes with 0.3 N/m spring constant would remove the cell from the surface. This is not related to the vitality of the cell as it happened within the first hour of removing cells from the incubator. Typically the cell would remain attached to the tip, requiring starting over with a new tip. Second, we have found that it is possible to actually enter the cytoplasm of the cell, keeping the cell alive. It is only because of our unique combination of fluorescence microscopy to assay cell vitality and in-situ calibrated capacitance standards that we are able to achieve this major milestone of an in-cell nanoelectrode with calibrated circuit connectivity from DC all the way up to 26 GHz. We describe this major advance next.

In Fig. 6, we show an image of a single cell with TMRE proving the cell is vital. Prior to bringing the tip into contact with the cell, we imaged the disks to calibrate the SMM (Fig. 6g). Then, we approached the cell from the top. After reaching the surface of the cell, we purposefully moved the tip down an additional 3 microns, to ensure that the tip was actually inside the cell. The microwave reflection was measured at different frequencies when the probe was inside the cell. The measurement is the first ever broadband calibrated electrical connected to the inside of a cell. The figure shows the capacitance from tip to ground changed as the tip was inserted into the cell. The calibrated capacitance is distinct from when the tip is outside the cell, and distinct from the calibration nano-disk capacitance as well as the stray capacitance. The cell cytoplasm contents are too soft to enable imaging inside of the cell, but in the future it may be possible to impale the cell at different locations, especially enabled by our integrated fluorescence microscope. In order to confirm definitively that the tip was inside the cell, at the end of the experiment, the tip was scanned from left to right and the cell was carried with it and disrupted (see video in Supplementary information 2), confirming it was inside the cell.

**Figure 6:**
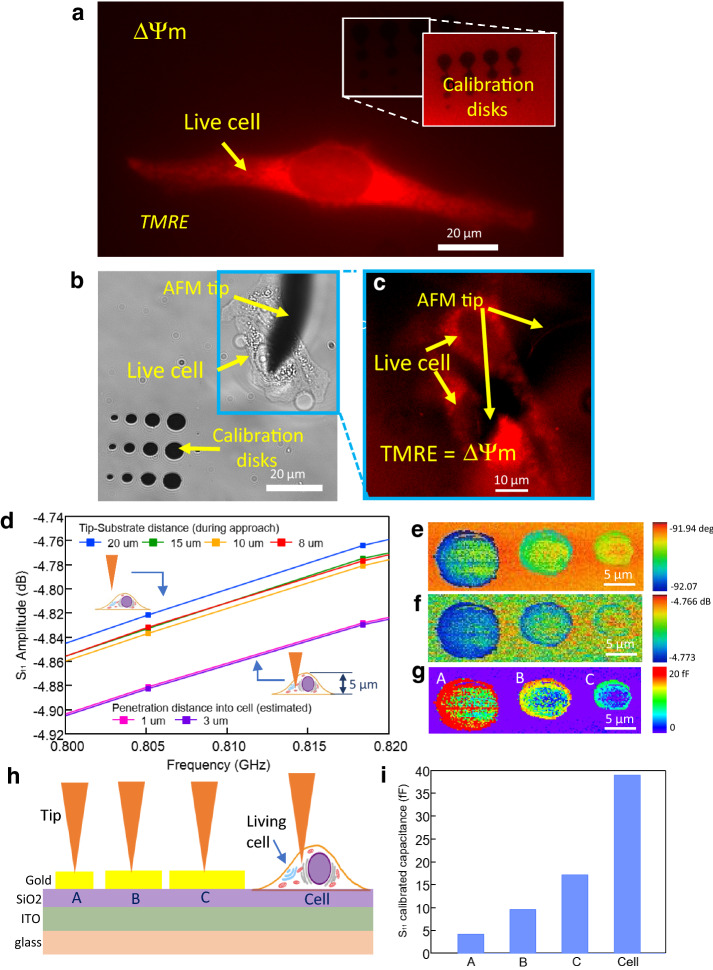
“Proof of life” imaging of vital cells with integrated calibration nano-disks. (**a**) TMRE image of a live HeLa cell. The TMRE fluorescence is proportional to the mitochondrial membrane potential ΔΨm. (**b**) Live HeLa cell with inserted nanoprobe, also showing integrated calibration nano-disks. (**c**) TMRE image of same HeLa cell, showing “proof of life” In b,c two cells are visible. (**d**) Amplitude of the complex reflection S_11_ at ~ 0.8 GHz for probe inside, outside cell. (**e**, **f**) SMM image of calibration nano-disks. (**g**) Calibrated capacitance image using calibration method presented in text. The variation of the capacitance density along a single calibration disk is due to impurities on the disk, which occasionally occurs in air as well, so is not related to the liquid imaging technique per se. (**h**, **i**) Cartoon and capacitance data for bare wafer (no capacitance), nano-disks, and interior of cell.

While the viability is a subject for future research, this shows a major advance in calibrated electronic imaging, and potentially sensing and actuation (Fig. 7), of the morphology and electronic activity of organelles within the cytoplasm. This enables future research on the effect of cell viability and biochemistry on scanning microwave microscopy images.

**Figure 7:**
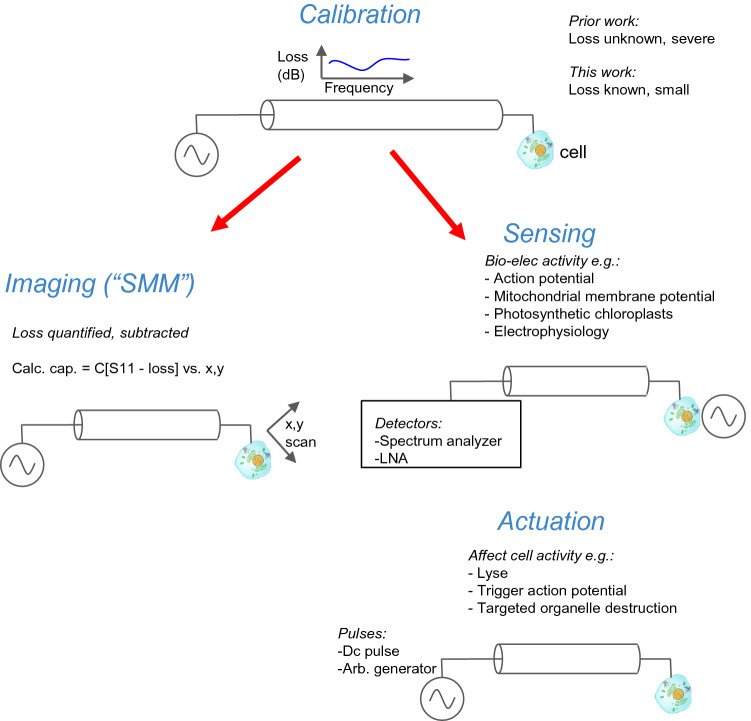
A broadband, calibrated interface to cells has more than just capacitance imaging applications. Other applications include sensing and actuation. All are enabled by the calibrated, broadband electrical interface to the interior of a living cell as demonstrated in this paper.

What is the ultimate limit of this technique? Although this platform has many potential applications, we now discuss one use case, that is capacitive imaging. The typical capacitance of a lipid bilayer is ~ µF/cm^2^. If we ask the effective area of a lipid bilayer with 5 fF of capacitance the answer is ~ 100 nm × 100 nm. Therefore, with this system, it should be possible to resolve, with non-invasive capacitive imaging alone, organelles as small as 100 nm, such as for example, mitochondria, chloroplasts, lysosomes, and the structure of the ER, as well as of course the nucleus. We note that, from the presented data (Fig. 5f, g), whether the method can be directly transferred to analyze intracellular organelles is not fully proven. However, we have already shown the ability to image vital isolated organelles such as mitochondria with fluorescence microscopy^22^. We aim in future work to extend our work on isolated organelles to vital organelles within the cell. We presented AFM topography images obtained simultaneously in Fig. 5f, g as Fig. 4 in Supplementary information 1, from which the cell nucleus area and cell membrane boundary can be resolved easily. The AFM probe being used in Fig. 5 has a radius lower than 20 nm, the scan area was 60 μm × 60 μm with 512 points /line, which makes each pixel theoretically around 117 nm × 117 nm. The lateral resolution in Fig. 5f, g is around 100–140 nm, zooming details of both AFM tapping mode amplitude and PNA phase cross-sectional profile on disk edge and cell boundary are presented in Fig. 5 in Supplementary information 1. To this end a combination of deep-subwavelength imaging technologies such as Airy scan and our SMM imaging should be compatible. We note that recently, Airy scan techniques have proven^28^ that mitochondria membrane potential is not uniform within the organelles, confirming our finding of this by indirect techniques in Ref.^29^ Furthermore, the Airy scan can image the individual cristae, so combined with nanoelectronic probing, although many challenges remain, this paper represents a significant step towards the final goal of electrical and optical probing of organelle activity in a living cell at the nanoscale.

What kind of the information on living cells and subcellular organelles would be extracted via the capacitance mapping? We can answer that question simply by comparing with optical microscopy. What kind of information on living cells and subcellular organelles would be extracted by measuring the scattering of light? The answer from a biophysical perspective is that the dielectric constant ε(ω) at the optical frequency causes scatting of light, which is measured in an optical microscope. This information is very useful to image because it gives structural information about the cell and organelles. In this work the principle is the same: the capacitance mapping measures the dielectric constant ε(ω) at the RF frequency, and gives structural information about the cell and organelles. However, it is a complementary technique as compared to optical methods to measure morphology that does not require fluorescence tagging and does not require extremely high illumination intensities. Thus, it can be thought of as a “kinder, gentler” nanoscale morphology imaging technique as compared with optical imaging.

We envision as one of many possible use cases imaging the morphology of mitochondria during apoptosis^4,30,31^. It is known the morphology of mitochondria inner structure changes during apoptosis, but the details are difficult to discern because most optical imaging causes damage due to the strong illumination intensity. Although we may not be able to resolve the individual cristae (to be determined by the final spatial resolution of the technique), the cristae density should be measurable and is known to change during apoptosis.

That being said, now that we have demonstrated a calibrated, high bandwidth electrical interface to the interior of the living cell, the potential is there for sensing of electrical activity of cellular components as well as actuating it (Fig. 7). We discuss these two in turn.

The typical method of sensing electrical activity is with TMRE, as we have used in this paper. However, there are several disadvantages, namely (1) the spatial resolution is limited by the optical system used (2) the illumination intensity can sometimes kill the cell or damage the mitochondria, and (3) the temporal resolution is limited to 100 ms when the spatial resolution is diffraction limited, and is even slower for sub-wavelength imaging techniques. We recently carried out a quantitative study of the temporal resolution of TMRE assuming every photon was captured and the only noise source was the Einstein shot noise of the photons, as well as in more realistic situations^32^. There, we found that the temporal resolution was at best 100 ms. In contrast, we found that nano-electronic sensors that the mitochondria were attached to (non-optimized, just as proof of concept) were over 10× faster. Therefore, in this paper we are bringing the nanoelectronic sensor to the mitochondria inside the cell, so there is a huge potential advantage in speed and sensitivity above and beyond TMRE. A full discussion of the drawbacks of TMRE imaging is beyond the scope of this work. While we have not proved that in this paper, we have taken the first significant step towards sensing electronic activity (not just capacitance mapping) in the interior of live, vital cells, whose vitality is proven using traditional (TMRE) methods. While many labs have impaled cells with pipettes and other probes, a key advance of this paper is that we have a broadband, calibrated, low loss, and quantified interface to the interior of a living cell.

Turning next to electronic actuation, just as electrical stimulation of neural cells trigger the action potential, it should be possible to electronically stimulate the interior of cells to affect function. Although the actual experiments will be the topic of a separate paper, we have already demonstrated (in unpublished data) electrical actuation of cell activity as measured with TMRE. Again, while many labs can impale cells, none have used nano-scale metal electrodes to actuate and simultaneously measure cellular electrical activity as we have done. The quantitative broadband nature of our system enables this with unprecedented bandwidth and precision.

In the future, one could even use the broadband nanoelectronic interface even to interrogate quantum sensors, or to scale the frequency up to THz, where spectroscopic signature information about molecular species may even be available.

The use case of capacitive imaging is demonstrated in this paper, but the potential applications are much broader in scope and significance. Since the electrical coupling all the way to the AFM metal tip is well calibrated all the way up to 26 GHz, even in liquid, the interface could have a variety of applications in interfacing life sciences to nano-electronics at the GHz regime, including for example nano-radar for non-invasive, synthetic aperture radar type imaging of the morphology of the cytoplasm under different physiological conditions (including pharmacological manipulation), non-invasive imaging of the morphology of electrically active sub-cellular organelles such as mitochondria and chloroplasts under different metabolic and life cycle conditions (including cell death), passive “listeners” i.e. RF receivers of electrical activity (including mitochondrial membrane potential fluctuations) at frequencies well above that of prior art, active, local sources of highly localized (nanoscale) electric fields at frequencies up to 20 GHz/ time scales down to 100 ps, potentially down to the length scale of single ion channels and the time scale of the traversal of single ions through channels, and even injection and mechanical manipulation, with potential localization activity within the cytoplasm of a living cell.

## Supplementary information

Supplementary information

Supplementary Video
