# Supplementary material for: An ultra-high bandwidth nano-electronic interface to the interior of living cells with integrated fluorescence readout of metabolic activity
Source: Sci Rep. 2020 Jul 1;10:10756. doi: 10.1038/s41598-020-67408-5 (PMC7329815; doi:10.1038/s41598-020-67408-5)
Supplement: Supplementary file 1 — Supplementary information [file 41598_2020_67408_MOESM1_ESM.pdf]

Supplementary Information

An ultra-high bandwidth nano-electronic interface to the interior of living cells with integrated fluorescence readout of metabolic activity

Dandan Ren[1], Zahra Nemati[2], Chia-Hung Lee[3], Jinfeng Li[4], Kamel Haddadi[5], Douglas C. Wallace[6], Peter J. Burke[1,2,3,7]

[1]Department of Electrical Engineering and Computer Science, University of California, Irvine, CA 92697, USA. [2]Department of Chemical Engineering and Materials Science, University of California, Irvine, California 92697, USA. [3]Department of Biomedical Engineering, University of California, Irvine, CA 92697, USA. [4]Department of Physics and Astronomy, University of California, Irvine, CA 92697, USA. [5]CNRS, UMR 8520, Institute of Electronics, Microelectronics and Nanotechnology (IEMN), University of Lille, 59000 Lille, France. [6]Center for Mitochondrial and Epigenomic Medicine, Children's Hospital of Philadelphia and Department of Pediatrics, Division of Human Genetics, University of Pennsylvania, Philadelphia, PA 19104, USA. [7]Chemical and Materials Physics program, University of California, Irvine, CA 92697, USA.

Corresponding Author: pburke@uci.edu

Supplementary materials

Fabrication

The fabrication process started with 0.33 mm transparent glass, which has a coated layer of ITO film (230±50 Å) on one side. ITO coated glasses were cleaned with acetone and Isopropyl Alcohol (IPA) under ultrasonic conditions for 10 min each, and then dried with air. A 100 nm thick SiO_2 layer was directly deposited onto ITO surface using E-beam evaporation at a rate of 1 Å/s, leaving undeposited exposed ITO spots at corners for welding of grounding metal wire. After cleaning with acetone and IPA once more, ~70 nm thick PMMA A2 was spin-coated (4000 RPM for 45 s) onto SiO2 surface. Immediately after spin coating of PMMA, the halfway substrates were baked at 180 ºC for 90 s. Using e-beam lithography (EBL), PMMA layer was exposed to e-beam (30 kV, 25 pA and 350 C/cm^2) to create circular capacitance patterns with a diameter of 1, 2, 3 and 4 µm. After EBL, the samples were developed in MIBK/IPA 1: 3 developer for 70 s. After developing, the samples were rinsed by IPA and dried under air flow. To further clean PMMA residues, the samples were treated under Oxygen plasma for 5 min. Then, 50 nm gold on top of 20 nm Ti was deposited using e-beam evaporation at a rate of 0.5 Å/s. Finally, a copper wire was welded onto the calibration standard by conductive Nickel paint to ground the micro-capacitors during imaging.

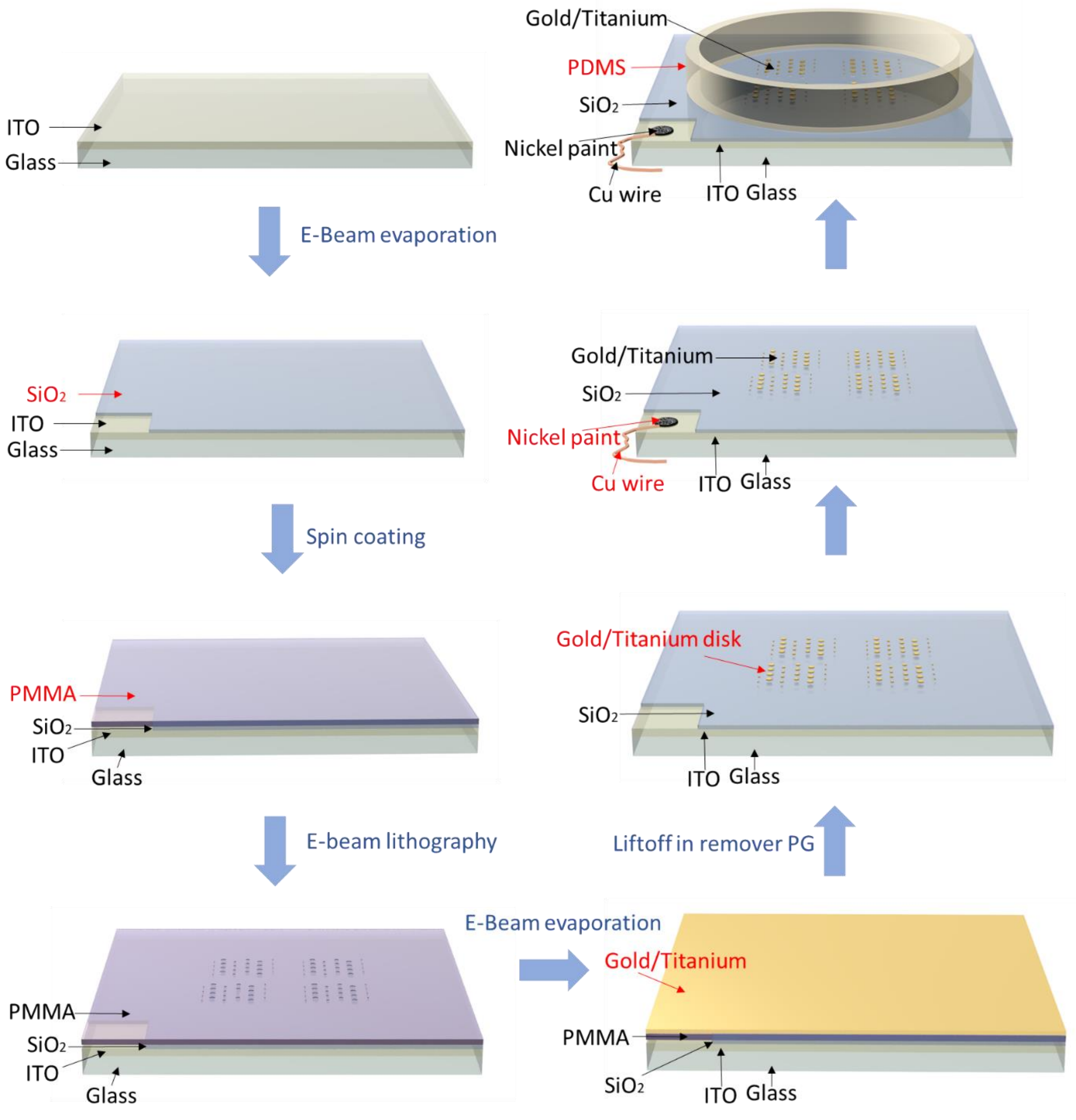

Figure 1: Process flow for on-wafer/on-petri dish nanoscale capacitance calibration standard fabrication.

Cell Culture & Imaging Process Flow

HeLa cell were purchased from ATCC®. HeLa are epithelial adherent cell lines derived from cervical cancer cells with a median size of 30-50 μm in diameter after attachment. The cells were cultured for 2-3 days in 75cm^2 tissue flasks in 37°C and 5% CO2 before being ready for experimentation. The protease trypsin was used to detach cells from the flask and seeded at 50-100k/ml on the calibration glass substrate. A thin PDMS chamber with the same perimeter as the substrate was designed and utilized to contain the media during seeding and the experiment. The chamber inner diameter corresponds to the AFM scanner head to prevent any disruptions during the scanning process. After 12-24 hours of incubation, the cells are tagged with either 1) TMRE potentiometric fluorescent dye (exc/em 549/574 nm) at 40nM for live analysis or 2) MitoTracker Green (exc/em 490/516 nm) at 100nM for fixed cells imaging. The cells were incubated with the lipophilic fluorophore for 30min to 1 hour. For live cell analysis, the TMRE+media solution is suctioned out and washed 2X and replaced with 1X PBS solution within the PDMS chamber. For fixed cell imaging, the MitoTracker green + media solution is washed out 2X with 1X PBS, fixed with proper volume of 4% formaldehyde for 15 minutes followed by 3 consecutive washes with 1X pre-warmed PBS solution. The sample is then either stored in -20°C for future experimentation or used immediately for scanning.

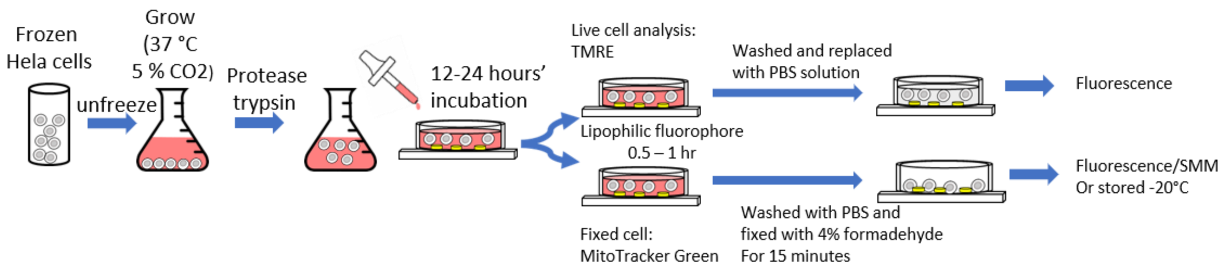

Figure 2: Workflow for cell culture.

22 GHz cell image

We plot microwave images at 22 GHz of fixed HeLa cells in Figure 3. This demonstrates the technique can be applied at these high frequencies.

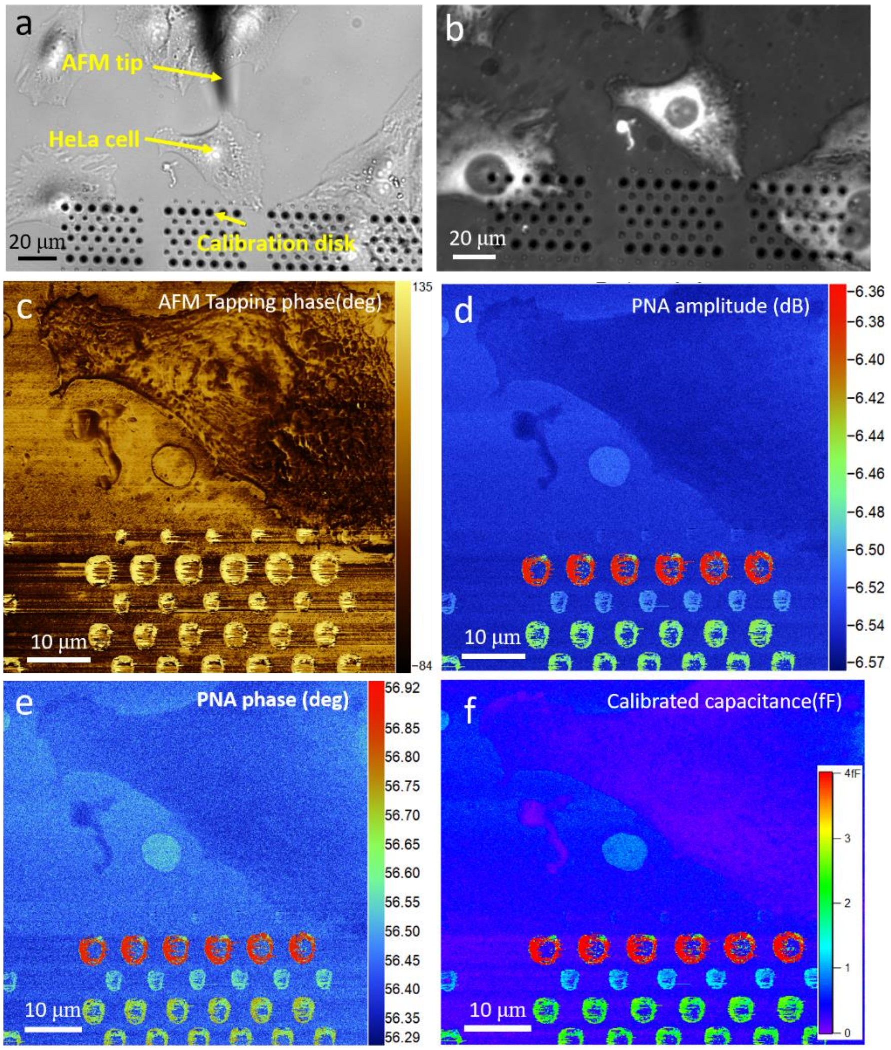

Figure 3: 22 GHz cell imaging. a) Brightfield. b) Fluorescence. c) AFM tapping mode phase. d,e) S_{11} image at 22 GHz.f) Calibrated capacitance.

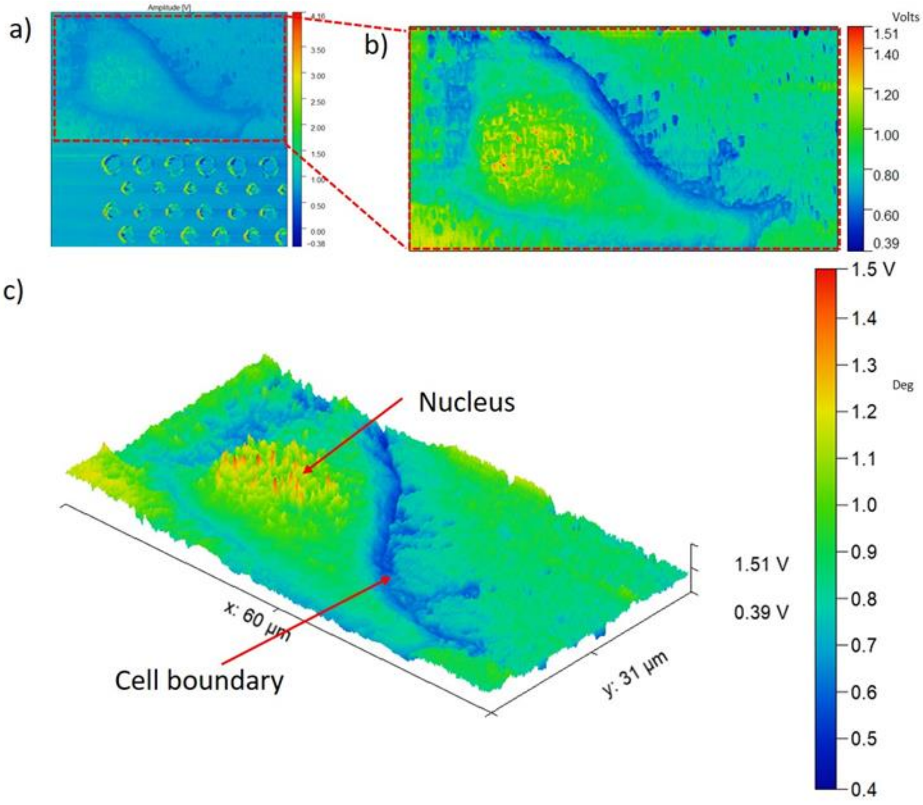

Figure 4 AFM tapping mode topography images corresponding to Fig 5f and 5g in main text. a, Tapping mode amplitude image. b, Zoom in on the cell of a. c, 3D view of b.

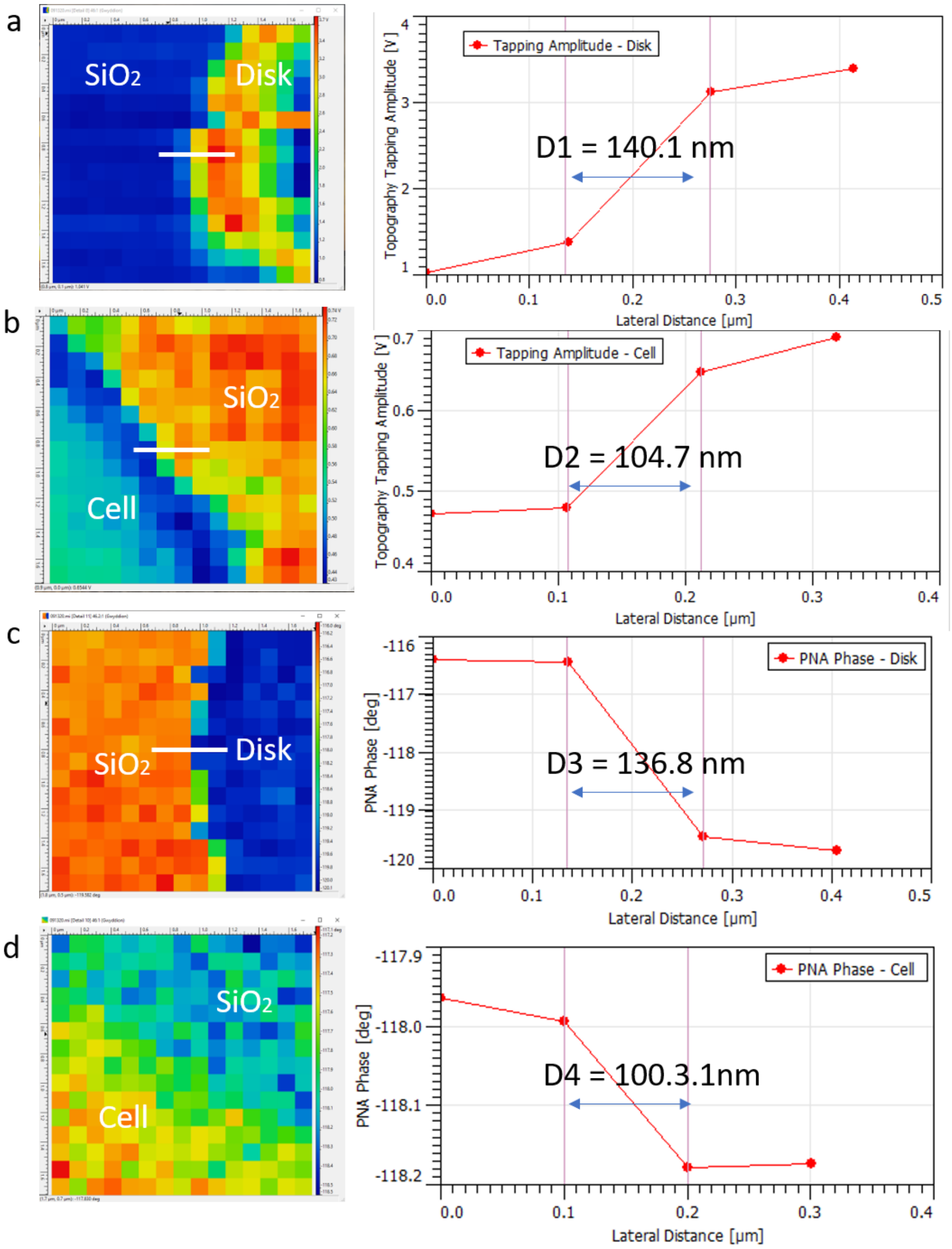

Figure 5: Zooming and cross-sectional images corresponding to Fig 5f and 5g in main text. a. AFM Tapping mode amplitude of gold disk edge. B, AFM Tapping mode amplitude of cell boundary. c, PNA phase of gold disk edge. d, PNA phase of cell boundary.

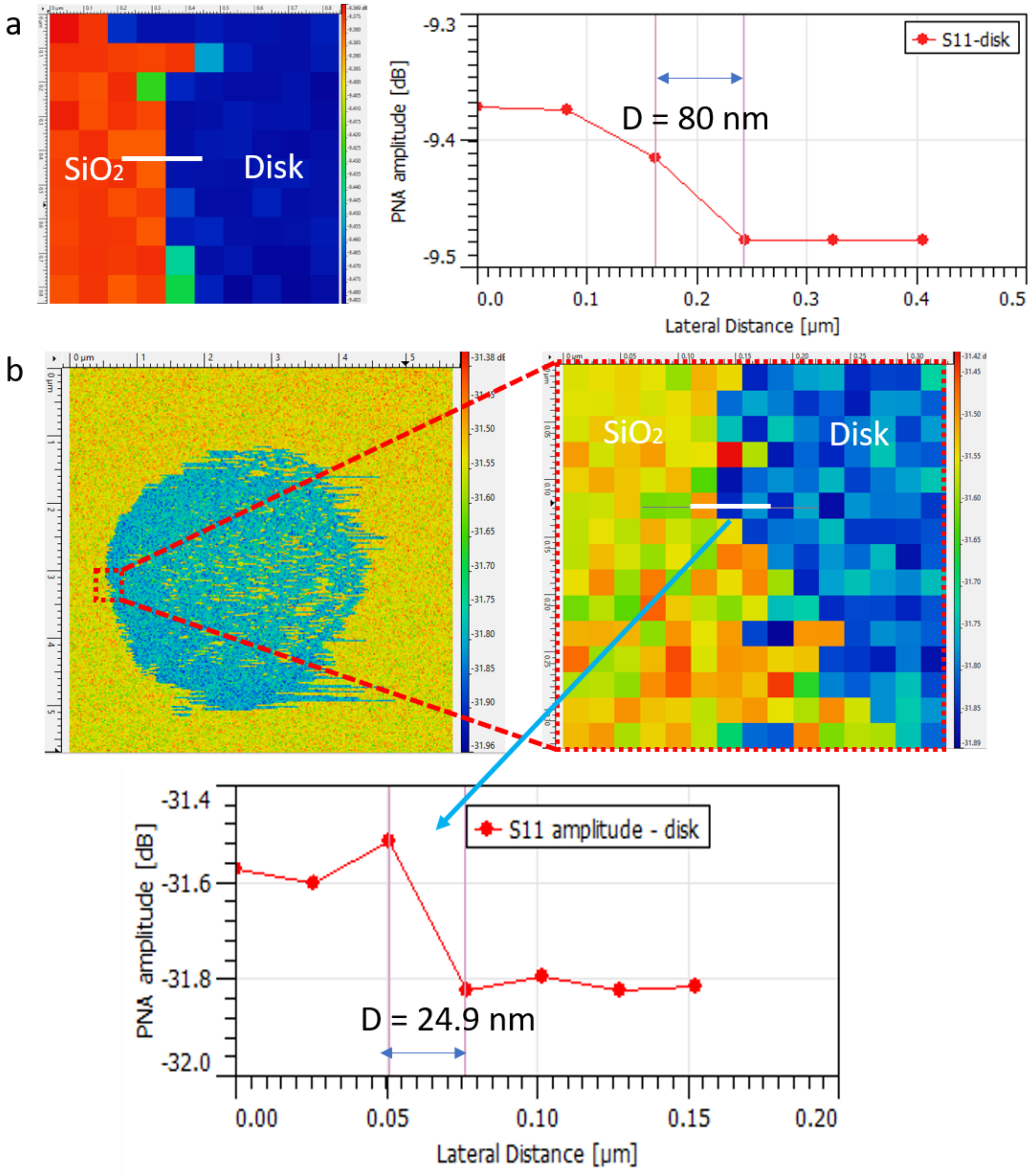

*Figure 6 SMM PNA amplitude resolution. a. Scan area 28 um, 256*256 pixels. b. Scan area 5.7um, 256 * 256 pixels*

Resonance vs. broadband

Keysight Technologies [13] has proposed the use of a shunt 50 Ω resistor to create a resonator using the last 10 cm or so of coax between the network analyzer and the AFM tip, as shown in Figure 7 below. The reason they proposed this is to bring the measured reflection coefficient closer to zero. However, we claim that this is not the parameter of interest to zeroth order, since what one cares about is the *change* in the measured reflection coefficient with a *change* in tip to ground impedance. The reason is that as one scans the tip, the change in the reflection coefficient is what is plotted in an image.

With that in mind, we calculate this quantity, $dS_{11measured}/dZ_{DUT}$, for both the resonator and non-resonator case, and show they are in fact the same. This means, to zeroth order, the resonator does not add any sensitivity. In fact, it only serves to confuse the measurement as the frequency dependence of $S_{11measured}$ is mostly determined by the resonator, not the device one want to measure.

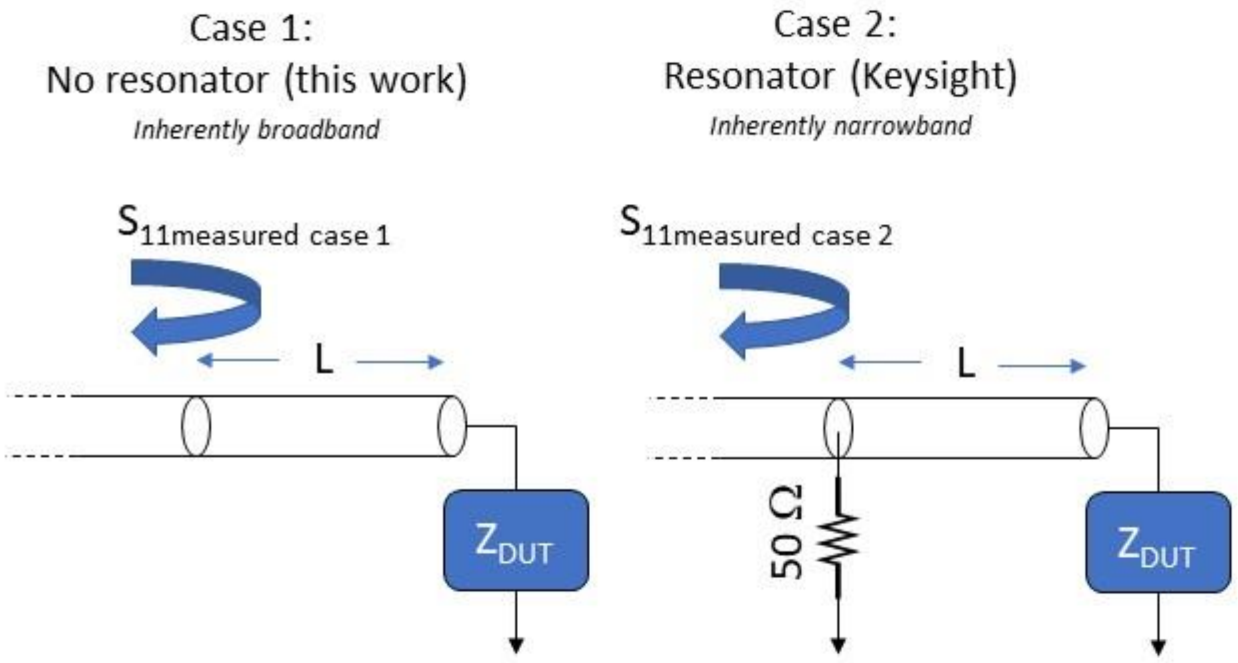

Figure 7: Broadband vs. resonant circuit coupling.

Case 1: No resonator

In this case, looking through a length L of an ideal transmission line (with characteristic impedance of 50 Ω) at a device under test with impedance Z_{DUT}, one has the following for the reflection coefficient measured looking in:

$$S_{11,measured} = e^{-i2kL}\frac{Z_{DUT} - 50\ \Omega}{Z_{DUT} + 50\ \Omega}$$

with k = 2π/λ the guided propagation constant and λ = v/f the guided wavelength of operation.

For the case of a high Z_{DUT}, i.e. the case of a small capacitance between the AFM tip and the ground plane, this can be Taylor expanded and shown to be approximately:

$$S_{11,measured\ (CASE\ 1)} \approx e^{-i2kL}\left(1 - 2\frac{50\ \Omega}{Z_{DUT}}\right)\left(1 - \frac{50\ \Omega}{Z_{DUT}}\right)$$

Therefore, the change in $S_{11measured}$ with Z_{DUT} is given by:

$$\frac{dS_{11,measured\ (CASE\ 1)}}{dZ_{DUT}} \approx 2\frac{50\ \Omega}{(Z_{DUT})^2}\frac{50\ \Omega}{(Z_{DUT})^2}$$

Case 2: With resonator

The resonator proposed, marketed, manufactured, and sold by Keysight Technologies is a 50 Ω to ground in parallel with a coaxial "tee", as show in Figure 7 above. Therefore, the resulting reflection coefficient can be calculated as the reflection coefficient of a 50 Ω load in parallel with the Z_{DUT} seen through a length L of transmission line, i.e.

$$S_{11,measured\ (CASE\ 2)} = \frac{\left(50\ \Omega \parallel Z_{effective}\right) - 50\ \Omega}{\left(50\ \Omega \parallel Z_{effective}\right) + 50\ \Omega}$$

where $Z_{effective}$ is Z_{DUT} seen through a length L of transmission line. $Z_{effective}$ can be shown to be:

$$Z_{effective} = 50\ \Omega \frac{1 - e^{-i2kL} S_{DUT}}{1 + e^{-i2kL} S_{DUT}}$$

where we have defined S_{DUT} as:

$$S_{DUT} \equiv \frac{Z_{DUT} - 50\ \Omega}{Z_{DUT} + 50\ \Omega}$$

One can Taylor expand the above also for case 2 for high Z_{DUT}, and one finds (on resonance where $e^{-i2kL} = 1$):

$$S_{11,measured\ (CASE\ 2,\ \ on\ resonance)} \approx \left(10 - 2\frac{50\ \Omega}{Z_{DUT}}\right)\left(1 - \frac{50\ \Omega}{Z_{DUT}}\right)$$

Therefore

$$\frac{dS_{11,measured\ (CASE\ 2,\ \ on\ resonance)}}{dZ_{DUT}} \approx 2\frac{50\ \Omega}{(Z_{DUT})^2}\frac{50\ \Omega}{(Z_{DUT})^2}$$

Which is identical to case 1. In fact, a similar conclusion applies off resonance, with only phase change in the derivative.

Therefore, the resonator reduces $S_{11measured}$ but does not change $dS_{11measured}/dZ_{DUT}$.

Phase shift of a S_{11} of a capacitive load vs. frequency

The reflection coefficient is given by: e

$$S_{11} = \frac{Z_{DUT} - 50\ \Omega}{Z_{DUT} + 50\ \Omega}$$

For a capacitive load, we have $Z_{DUT} = 1/(j\omega C)$. For small C (large Z_{DUT}), this can be Taylor expanded in 50/ Z_{DUT} to give:

$$S_{11} \approx 1 - \frac{100\ \Omega}{Z_{DUT}} = 1 - j100\omega C$$

This gives:

$$tan(\theta) = \frac{Imag(S_{11})}{Real(S_{11})} = -j\omega 100C$$

giving $\Delta\theta = -\text{atan}\,(\omega 2 \times 50C)$.

List of live cell imaging experiments

Cell type	Condition	Fluorescent Tag	Substrate	#times repeated
Hela	mostly dried	TMRE	Glass	1
Hela	liquid	TMRE	Glass	1
Hela	liquid	TMRE	Glass	1
Hela	liquid	TMRE	Glass	1
Hela	liquid	TMRE	Glass	2
Hela	liquid	TMRE	Glass + gold disc	3
Hela	fixed/liquid	MTG	Glass + gold disc	2
Hela	fixed/dry	MTG	Glass + gold disc	4-5 times
Hela	fixed/dry	MTG	Glass + gold disc	5
Hela	fixed/dry	MTG	Glass + gold disc	5-6 times
Hela	fixed/dry	MTG	Glass + gold disc	3 times
Hela	liquid	MTG	Glass+gelatin	1-2 times
gelatin + discs	dry		Glass gold disc + gelatin	2-3 times
L6 myoblasts	liquid	MTG	Glass	2-3 times
L6 myoblasts	liquid	MTG	PET+ITO	2
L6 myoblasts	liquid	MTG	PET+ITO	2
Fibroblasts	liquid	MTG	PET+ITO	1
L6 myoblasts	half-dry	MTG	PET+ITO	2
L6 myoblasts	liquid	MTG	PET+ITO	2
L6 myoblasts	liquid	TMRE	Glass+gold disc	1
L6 myoblasts	liquid	TMRE	Glass+gold disc	1
L6 myoblasts	liquid	TMRE	Glass+gold disc	3 times
L6 myoblasts	liquid	TMRE	PET+ITO	-
Fibroblasts	liquid	TMRE	Glass+gold disc	
L6 myoblasts	liquid	TMRE	Glass+gold disc	2-3
Fibroblasts	half-dry	TMRE	Glass+gold disc	3-4 times
L6 myoblasts	liquid	TMRE	Glass+gold disc	3
L6 myoblasts	liquid	TMRE	Glass+gold disc	2
Hela	liquid	TMRE	Glass+gold disc	1

The table reports data from a total of 67 experiments over the course of several months on single cell scanning microwave microscopy. In about half the experiments, a fresh (sharp) tip was used, and in about half the experiments a more worn out, dull tip was used. The experiments were performed on multiple cell lines adhered to various types of substrates in various environments as outlined in the table.

Initially HeLa epithelial cancer cell lines were used for SMM imaging. HeLa were cultured on a variety of substrates including glass, ITO (indium tin oxide) coated glass, plastic coverslips, gold discs on glass, and poly-L-lysine and gelatin coated glass substrates. The ITO and gold discs were utilized as calibration standards simultaneously with fluorescent imaging while poly-L-lysine and gelatin as immobilizing agents. However, none of the above substrates allowed the cells to remain intact during AFM/SMM imaging process, which lead to cell movement and eventual detachment and probe contamination, leading to premature termination of the experiment. Afterwards, we changed the cell lines from HeLa to a more robust L6 myoblast as well as fibroblast (skin) cell lines. Similar substrates and conditions were utilized for L6 and fibroblasts, however similar to HeLa, they also moved and detached during the scanning process. Furthermore, numerous AFM software parameters (i.e. I/P gain, scanner approach techniques, etc) were modulated and adjusted throughout the experiments to control for scanning factors that may have contributed the conditions that dislodge the live cells from the substrates.

We were successful in single cell SMM imaging with cell fixation via paraformaldehyde solution. This process would stiffen the plasma membrane enough, enabling us to obtain high resolution SMM images in liquid. The problem, however, is that due to the covalent cross linking between the molecules in the cell the fixation method effectively stops all cellular functions pertinent to survival, thereby eliminating the measurement of mitochondrial TMRE signal, solely present in live and functioning cells.

Complete response spectrum of sample mount

In Figure 8, we plot the measured S_{11} magnitude vs. frequency looking through about 12 inches of coaxial cable at the tip. The roundtrip loss is less the 10 dB (5 dB each way) for frequencies up to about 15 GHz. From 15-26.6 GHz, the losses become as large as 15 dB on average (7.5 dB each way) with increasing frequency dependence due to standing waves set up by non-ideal connectors and non-ideal coax to tip transition.

The conclusion is that coupling to the tip is broadband up to 26.5 GHz, with non-idealities in the system becoming more pronounced as the frequency increases.

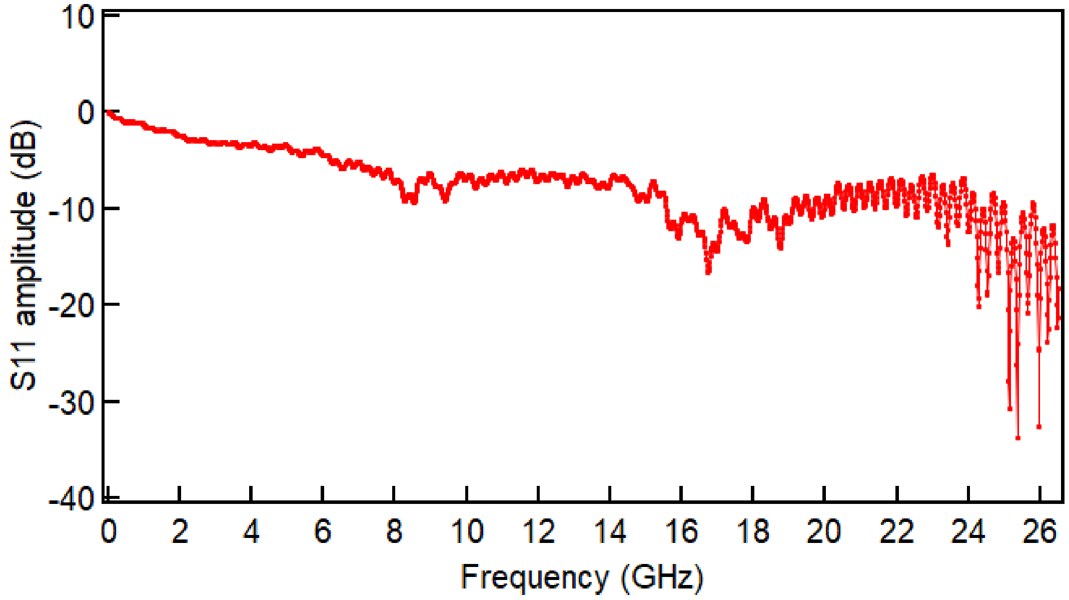

Figure 8: S_{11} spectrum of at the end of a hanging tip in air after PNA calibrated to 12 inch cable from the network analyzer.

Note added in press: While this paper was in press, a similar, complementary paper appeared (Jin, X., *et al*, *IEEE Trans. on Mic. Th. and Tech.* 2019, 67, 5438–5445). Our work is different in that it 1) is broadband 2) Has an integrated fluorescence microscope 3) has an integrated on chip/on petri dish calibration standard 4) uses TMRE as "proof of life" on the ACTUAL cell being measured, in real time, 5) provides interface into the inside of the cell. In sum our system is still the only demonstration of an integrated optical and SMM system which can provide "proof of life" on the actual cell being imaged.
